# Competition among steric vs resonance forces and conformational switchover: possible pseudo-Jahn-Teller effects and saturation of hyperpolarizabilities in Brooker’s zwitterions

**DOI:** 10.1007/s00894-026-07040-8

**Published:** 2026-11-03

**Authors:** Divya Pant, Bharati Bamana, Sanyasi Sitha

**Affiliations:** Department of Chemical Sciences, University of Johannesburg, Auckland Park, PO Box 524, Johannesburg, 2006 South Africa

**Keywords:** Nonlinear optics, Brooker’s zwitterions, Hyperpolarizability, Conformational switch, Saturation limit

## Abstract

**Context:**

This work reports structure, linear, and nonlinear optical properties of pyridinium imidazolate-based Brooker’s types of zwitterions, with benzene ring(s) as repeating bridges (no bridge to 4-benzene rings). Instead of the expected planar conformations, significant conformational switching was observed for the molecules with 3- and 4-benzene rings as bridges. Such conformational flipping showed a saturation limit for the nonlinear optical response with 3-benzene rings as bridges (CAM-B3LYP: *β* = 3760.4 × 10^−30^ esu and |*μβ*| close to 0.18 million × 10^−48^ esu—*among the largest values reported in literature till date*). Thus, an early saturation was observed (with 3-benzene rings as bridges) before pseudo-Jahn-Teller distortion (observed with 4-benzene rings as bridges). This report shows how the gyratory nature of the inter-ring junctions plays significant roles in controlling intramolecular interactions and response properties. This work also shows that structural topography plays vital role in efficient chromophore design. This work reports molecules with extremely large dipole moments, polarizabilities, and molecules also with absorptions in the IR regions of the electromagnetic spectrum (maybe useful to solve transparency-tradeoff problem of nonlinear optics).

**Methods:**

Geometry optimizations and further computations of all the molecules were performed with Gaussian 09 program, with HF, B3LYP, and CAM-B3LYP methodologies combined with 6-31G(d,p) basis set. For all molecules, computations of adiabatic excitations were carried out using TDDFT and TDHF approaches. For the computations of the response properties (like the nonlinear optical responses), CPHF and CPKS approaches were used. Population studies were carried out to visualize molecular orbitals and electrostatic potential maps. NBO computations were carried out to account atom hybridization and orbital interaction energies.

**Supplementary Information:**

The online version contains supplementary material available at https://doi.org/10.1007/s00894-026-07040-8.

## Highlights

- Conformational switching to increasingly twisted conformations observed for Brooker’s zwitterions.
- Dipole moment and polarizabilities showed increasing trends; and electronic and optical gaps showed decreasing trends, with increasing bridge lengths.
- Only molecular 1^st^ hyperpolarizability (β) showed early saturation limit, with 3-benzene rings as the bridge.
- Observed β  = 3760.4 × 10^-30^ esu, with |μ| = 0.18 million × 10^-48^ esu is one among the large values of reported in the literature.
- IR absorptions of the zwitterions may be helpful to overcome transparency trade-off problem of nonlinear optics.

**Graphical Abstract:**

**Supplementary Information:**

The online version contains supplementary material available at https://doi.org/10.1007/s00894-026-07040-8.

## Graphical abstract

**Figure:**
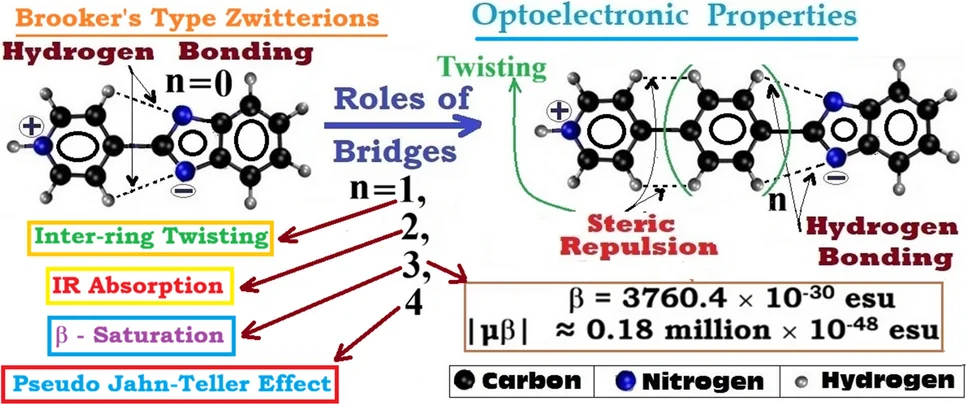

## Introduction

**Scheme 1:**
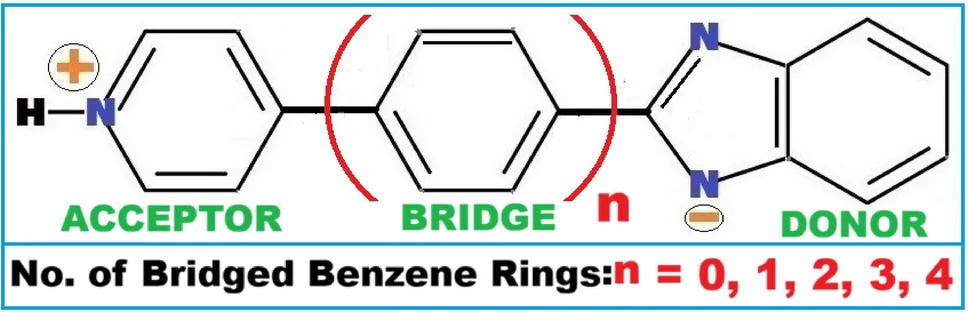
Brooker’s type of donor-bridge-acceptor-based zwitterions with varied number of *p*-phenylene (benzene ring) bridges

In organic chemistry, zwitterions are an unconventional class of organic molecules and commonly also known as inner salts or dipolar ions, as they generally exhibit charge-separated ground state configurations [1]. Two important segments, the cationic (+vely charged segment, which can act as an acceptor, “A”) and the anionic (−vely charged segment, which can act as a donor, “D”) subunits are generally linked to each other through covalent bonding, to constitute a charge neutral single molecule [2]. Owing to such interesting characteristics (highly polar natures), in the past zwitterions were investigated in various fields [3–7]. It is to be noted here that a Clarivate Science Citation Index Expanded (Clarivate SCIE) search can locate thousands of scientific articles on or about zwitterionic systems, with applications in the areas of chemistry, physics, and material science. Moreover, due to their dipolar nature, such molecules are classical D/A dyads (D and A are connected via π-linkers, to form D-π-A type of push-pull molecules) and thus were also studied as suitable functional molecular materials in the past [6, 8–22]. Among various applications as functional materials, one important area of application is functional molecular materials for nonlinear optics (NLO), and thus zwitterions were also extensively studied in the past as interesting materials for NLO [23–47]. In recent times, our group also extensively studied many kinds of zwitterionic systems and suggested them as potential NLO active functional molecular chromophores, with large molecular first hyperpolarizabilities and ground state dipole moments [48–55]. In our previous systematic studies on various classes of zwitterions for NLO, we have observed that, when benzene ring acts as a bridge (B) between the D and A, often enhanced hyperpolarizability were observed. Besides this crucial advantage associated with benzene ring as the bridge, benzene bridge is also capable of providing conjugation path as well as structural rigidity to the molecular chromophore. Hence, in this work, we have now investigated few zwitterionic molecules with varied number of benzene rings as the bridges (sequentially increasing the bridge length), placed in the charge transfer path between the D and A. By placing the benzene rings in the bridge path through *p*-phenylene type of linkage, five molecules were constructed and investigated for this study. A schematic diagram for the investigated zwitterions is presented in Scheme 1.

All the molecules considered here are of Brooker’s type zwitterions [56, 57] and are also inspired by similar types of zwitterions whose synthesis is already reported in the past [58]. Like the previously reported synthesized molecules, here also we have considered pyridinium as the acceptor (A) and benzimidazolate as the donor (D) and constructed five zwitterions in Brooker’s bonding mode [58]. Through *p*-phenylene bridge(s), a conjugated linear path was provided between D and A. As a result, these molecules can be expected to be of linearly extended conjugated systems, and at the same time due to multiple repulsive (destabilizing) steric repulsions as well as stabilizing hydrogen bonding interactions, they can also be expected to show interesting structure-property correlations through molecular rotational conformations [22]. This is the main motivation of the current work, and with this we are interested to see how these interesting behaviors affect NLO activities in this series of molecules. With this aim, we have carried out a detailed computational investigation for all five zwitterionic chromophores and studied their linear as well as nonlinear optical response properties, and some other important molecular electronic properties also. Interesting trends in functional molecular properties, conformational switchover, activity saturations, etc. were observed, also investigated and reported.

## Computational methods

All five molecules were fully optimized (without any symmetry restriction to allow inter-ring rotational freedoms) with HF [59], B3LYP [60, 61], and CAM-B3LYP [62] methodologies using Gaussian 09 Quantum Chemistry software [63]. Choice of methodologies was based on recent computational research activities by many researchers working in the field of nonlinear optics. Between HF and B3LYP, while the former method lacks electron correlations, B3LYP accounts for such correlations. Comparison between them will provide the roles of electron correlations on molecular conformations and response properties. Moreover, a recent study also suggests better performance of HF over DFT-based methodologies, especially for similar kinds of zwitterionic molecules [64]; thus, such a comparison will be interesting. Now, between B3LYP and CAM-B3LYP, the latter one includes additional long-range corrections, and good performance long-range corrected methodologies for predicting nonlinear optical behaviors were reported in earlier literature [65–72]. Hence, comparison between these two will be able to account for the effects of long-range corrections and their impact on molecular conformations as well as response properties. For all three methodologies, Pople’s basis set 6-31G(d,p) was used for all computations (for *n* = 3 and 4 cases—Scheme 1, optimization and computations of molecular properties were also carried out with 6–31++G(d,p) basis set to account of impact of diffuse functions). From the analysis of vibrational frequencies, all five molecules were confirmed to be true minima (showed all positive frequencies or no negative Eigen values in the Hessian—first six harmonic frequencies for all the molecules are provided in supporting information). After full optimizations, using the optimized molecular geometries, in respective methodologies, further computations were carried out at the same level of theory. Frontier molecular orbitals like HOMO (Highest Occupied Molecular Orbital) and LUMO (Lowest Unoccupied Molecular Orbital) and their energies, molecular electrostatic potential (MEP or ESP) surfaces [73, 74], dipole moments, etc. were obtained using their respective optimized geometries. Time-dependent (TD: TDHF and TDDFT) [75, 76] computations were performed to investigate adiabatic excitations and linear optical properties of respective molecules. Then, other response properties like the polarizabilities and hyperpolarizabilities were computed for these five molecules using the coupled perturbed (CP: CPHF and CPDFT/CPKS) methodologies [77, 78]. Then using following well-known equations, already reported in the literature, functional molecular properties were computed [79–83] (conversion factors: *α*: 1 a.u. = 0.1482 × 10^−24^ esu and *β*: 1 a.u. = 8.6393 × 10^−33^ esu).

Using the three dipole components (*μ*_*x*_, *μ*_*y*_, and *μ*_*z*_), total dipole moments (*μ*_tot_) were computed using Eq. 1.

1 $${\mu}_{\mathrm{t}\mathrm{o}\mathrm{t}}=\sqrt{{\mu}_{x}^{2}+{\mu}_{y}^{2}+{\mu}_{z}^{2}}$$

The average polarizabilities $\langle \alpha \rangle$ were computed using three components, *α*_*xx*_, *α*_*yy*_, and *α*_*zz*_, in Eq. 2.

2 $$\langle \alpha \rangle =\frac{{\alpha}_{xx}+{\alpha}_{yy}+{\alpha}_{zz}}{3}$$

Using the components of molecular hyperpolarizability, total 1 st hyperpolarizability (*β*_tot_ also known as *β*_0_) and |*μβ*| were computed using Eqs. 3 and 4 respectively.

3 $${\beta}_{0}=\sqrt[2]{{\beta}_{x}^{2}+{\beta}_{y}^{2}+{\beta}_{z}^{2}}$$

4 $$\left|\mu \beta \right|=\sum_{i}^{x,y,z}{\mu}_{i}{\beta}_{i}={\mu}_{x}{\beta}_{x}+{\mu}_{y}{\beta}_{y}+{\mu}_{z}{\beta}_{z}$$

Here, $${\beta}_{x}={\beta}_{xxx}+{\beta}_{xyy}+{\beta}_{xzz}$$, $${\beta}_{y}={\beta}_{yyy}+{\beta}_{yxx}+{\beta}_{yzz}$$, and $${\beta}_{z}={\beta}_{zzz}+{\beta}_{zxx}+{\beta}_{zyy}$$.

## Result and discussion

**Fig. 1:**
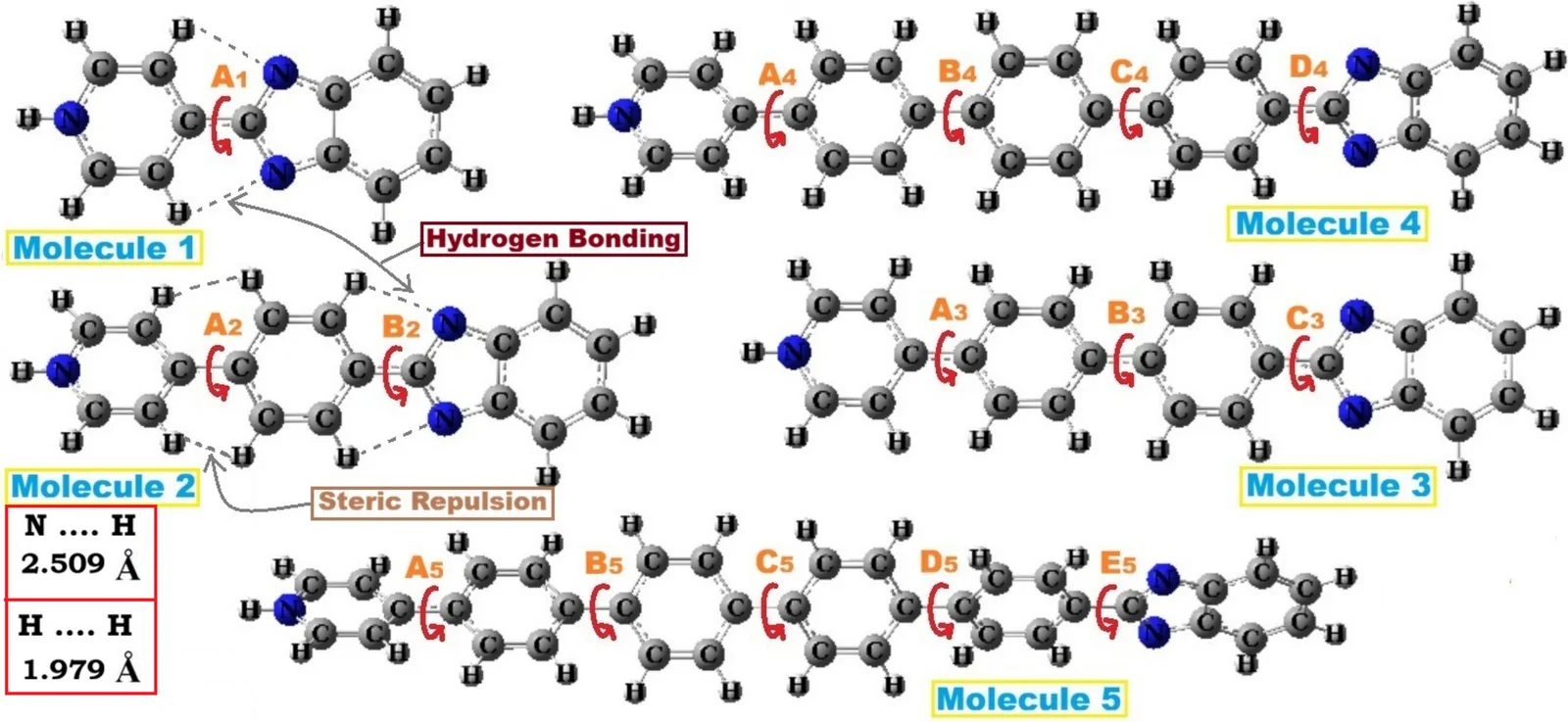
Molecular geometries of Molecules **1**–**5**, in their minimum energy conformations (from CAM-B3LYP methodology)

As discussed in the computational section, all the five molecules were fully optimized without imposing any symmetry restrictions during the optimization processes. Minimum energy conformations of all the five molecules (*n* = 0: Molecule **1**, *n* = 1: Molecule **2**, *n* = 2: Molecule **3**, *n* = 3: Molecule **4**, and *n* = 4: Molecule **5**) are shown in Fig. 1. As saturation of hyperpolarizability response was observed (discussed in the latter section), hence no more extended bridges were considered for investigations.

### Minimum energy conformations

Molecule 1 being the D-A directly connected (without the bridge or *n* = 0) molecule, it has only one inter-ring junction. As expected, due to the presence of two stabilizing hydrogen bonding (H-bond) interactions (Fig. 1), the observed conformation was found to be fully planar (A_1_ = 0.0°) in all methodologies. Now, Molecule 2 was found to be in slightly twisted conformation (CAM-B3LYP: A_2_ = 6.1° and B_2_ = 0.1°; HF: A_2_ = 9.1° and B_2_ = 0.1°; and B3LYP: A_2_ = 2.6° and B_2_ = 0.1°). At the pyridinium side (A) junction (twist angle—A_2_), twisting was observed compared to the benzimidazolate (D) side junction (twist angle—B_2_), which was found to be in almost planar conformation. Due to the presence of two repulsive H….H steric repulsions (H-H-steric repulsion) arising between pyridinium-benzene (interaction between two 6-membered aromatic rings), a slight twisting was observed for A_2_ (competition between destabilizing steric repulsion and stabilizing mesomeric resonance, the former one can be expected to slightly overpower over the latter force [22]—to be noted here that above is qualitative interpretation only). At the same time, at the other junction (B_2_), due to the presence of two stabilizing H-bond interactions, it was found to be in planar conformation.

Now for Molecules 3–5, besides the pyridinium-benzene and benzene-benzimidazolate junctions, additional junctions were observed in-between the bridge benzene rings (benzene-benzene junctions). Thus, such additional benzene-benzene junctions being capable of contributing additional H-H-steric repulsions (qualitatively from the structural features one can easily expect), more and more twisted conformations for Molecules 3–5 can be expected. Due to observed more or less similar trends in methodologies, for Molecules 3–5 only the CAM-B3LYP geometries are discussed here. It is also to be noted here, with respect to methodologies, while HF predicted bigger inter-ring twist angles, at the same time B3LYP showed lower values and CAM-B3LYP showed intermediate values between HF and B3LYP. Minimum energy conformation for Molecule 3 showed twisted conformation with twist angles as A_3_ = 7.0°, B_3_ = 14.8°, and C_3_ = 0.1° (CAM-B3LYP). While C_3_-junction was found to be planar, A_3_-junction was found to be slightly more twisted compared to Molecule 2 (almost retention of similar twist angle) and the B_3_-junction was found to be more twisted than A_3_. Minimum energy conformation for Molecule 4 showed enhanced inter-ring twist angles (CAM-B3LYP: A_4_ = 13.1°, B_4_ = 22.5°, C_4_ = 24.2°, and D_4_ = 0.3°) compared to Molecule 3. In other words, a significant conformational switching was observed for Molecule 4 (not only for B_4_ and C_4_, but also for A_4_) compared to Molecule 3. From these observations, qualitatively one can interpret that in the competition between destabilizing steric repulsion and stabilizing resonance forces, now the repulsive forces may be overpowering over resonance forces, thus extending their influences even to the A_4_-junction [22]. Optimized geometry of Molecule 4, in CAM-B3LYP/6–31++G(d,p), showed very small differences (A_4_ = 13.5°, B_4_ = 23.1°, C_4_ = 24.9°, and D_4_ = 0.3°), compared to that of 6-31G(d,p) optimized geometry. As expected, minimum energy conformation for Molecule 5 showed even larger inter-ring junction twist angles compared to Molecule 4 (for Molecule 5, CAM-B3LYP: A_5_ = 19.0°, B_5_ = 29.9°, C_5_ = 32.3°, D_5_ = 30.8°, and E_5_ = 0.1°). A significant increase in the A_5_ value was observed compared to A_4_. In CAM-B3LYP/6–31++G(d,p), optimized geometry of Molecule 5 also showed very small differences (A_5_ = 19.0°, B_5_ = 30.4°, C_5_ = 33.6°, D_5_ = 32.3°, and E_5_ = 0.3°), compared to its 6-31G(d,p) optimized geometry. With these observations, it can be predicted that Molecule 4 and/or 5 can be expected to show some kind of switching in the projected trends of the properties.

### Electronic properties

Electronic properties like dipole moments (*μ*) and frontier molecular orbitals, HOMO and LUMO, and their respective energies *E*_*H*_ and *E*_*L*_ were computed using all three methodologies and tabulated in Table 1.

**Table 1:** Dipole moments (major component, *μ*_*x*_ and total dipole moment, *μ*_*T*_) in Debye and orbital energies (*E*_*H*_ and *E*_*L*_) and HOMO-LUMO gap (HLG) in eV, for Molecules 1–5, computed using HF, B3LYP, and CAM-B3LYP methodologies

Molecule #	Methodologies	*μ*_*x*_	*μ*_*T*_	*E*_*H*_	*E*_*L*_	HLG
1	HF	10.8	10.8	−6.6	0.9	7.5
	B3LYP	9.6	9.6	−4.9	−2.2	2.7
	CAM-B3LYP	10.3	10.3	−6.2	−1.1	5.1
2	HF	21.9	21.9	−5.6	0.01	5.6
	B3LYP	17.4	17.4	−4.5	−2.7	1.8
	CAM-B3LYP	19.6	19.6	−5.4	−1.9	3.5
3	HF	43.6	43.6	−4.8	−0.9	3.9
	B3LYP	25.8	25.8	−4.2	−3.1	1.1
	CAM-B3LYP	31.8	31.8	−4.9	−2.5	2.4
4	HF	68.2	68.2	−4.2	−1.5	2.7
	B3LYP	34.5	34.5	−3.9	−3.4	0.5
	CAM-B3LYP	48.1	48.1	−4.4	−3.1	1.3
5	HF	89.9	89.9	−3.9	−1.8	2.1
	B3LYP	42.7	42.7	−3.8	−3.5	0.3
	CAM-B3LYP	62.8	62.8	−4.0	−3.3	0.7

As expected, an increase in dipole moments was observed on moving from Molecule 1 (CAM-B3LYP: 10.7 D) to Molecule 5 (CAM-B3LYP: 62.8 D—very large value). In general, HF predicted the largest, B3LYP predicted smallest (except Molecule 1—all three methodologies predicted nearly similar values), and CAM-B3LYP predicted the intermediate values of dipole moments in respective molecules. It is to be noted that *μ*_*x*_ (major component and oriented along the molecular rotational axis) and *μ*_*T*_ were found to be quantitatively almost indifferent from each other. In CAM-B3LYP/6–31++G(d,p), slightly enhanced dipole moments were observed (Molecule 4: 50.4 D and Molecule 5: 66.1 D) compared to 6-31G(d,p) dipole moments as shown in Table 1.

**Fig. 2:**
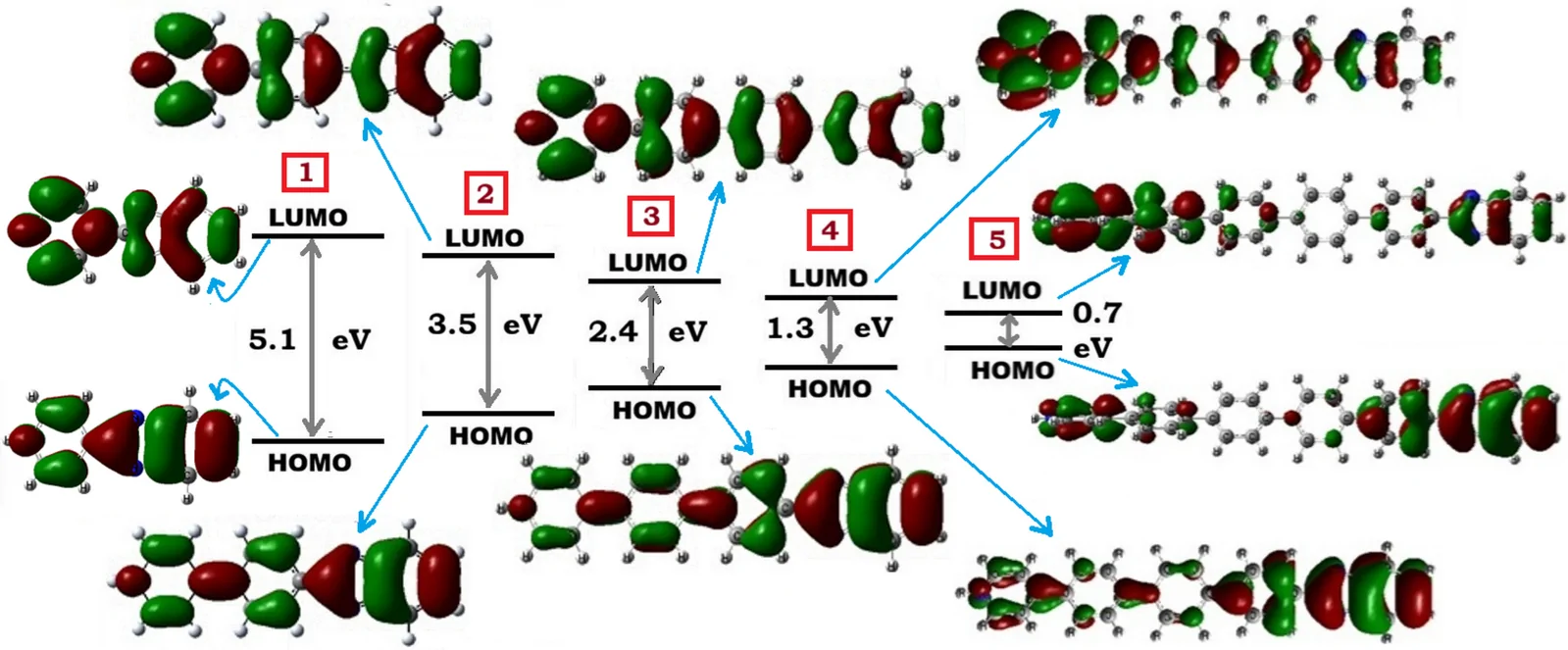
HOMO-LUMO orbital pictures and HLG (HOMO-LUMO gap) values (in eV) for Molecules 1–5, from CAM-B3LYP computations (Iso values: 0.02)

HOMO and LUMO for Molecules 1–5 were generated (from CAM-B3LYP) and are shown in Fig. 2. Also, for all the five molecules, ESP maps are provided in the supporting information (Fig. S1). As ESP has no direct implication on the nonlinear optical properties, hence they are not discussed in detail here. Nevertheless, all these five molecules showed distinctive positive potential regions around their respective pyridinium sites and regions of negative potential sites around their respective benzimidazolate parts, a characteristic can be directly attributed to their zwitterion natures. Analysis of population densities indicates that for Molecules 1–4, both the HOMOs and LUMOs were found to be delocalized (slightly lesser densities in the central region were observed for Molecule 4), and at the same time for Molecule 5, in the central region these two orbitals were found to be with relatively depleted densities.

The orbital pictures and HLG values from CAM-B3LYP computations for Molecules 1–5 are shown in Fig. 2. As can be seen from Fig. 2, largest HLG was observed for Molecule 1 (5.1 eV) and as expected for extended conjugated system, HLG showed decreasing trends when moving towards Molecule 5 (showed the lowest HLG of 0.7 eV). As expected, in moving from Molecule 1 to 5 successively LUMO was found to be getting stabilized, and HOMO was getting destabilized. As a result, a decreasing trend in the HLG values was observed. HLG, also known as electronic gap, the observed decreasing trend can be expected to play significant roles in the optical absorption spectra as well as many other electronic properties. Among three methodologies (Table 1), HF showed larger and B3LYP showed lower values of HLGs than CAM-B3LYP methodology. However, all the three methodologies showed similar trends in HLGs on moving from Molecule 1 to 5. From Table 1, compared to CAM-B3LYP, HF predicted destabilized LUMO and stabilized HOMO, resulting in increasing values of HLGs for these molecules. Similarly, compared to CAM-B3LYP, destabilized HOMO and stabilized LUMO were observed for B3LYP, resulting in reduced values of HLGs for these molecules.

### Optical properties

TD vertical excitation computations were carried out with the optimized geometries of all five molecules in all three methodologies. Computed optical absorption properties of Molecules 1–5, from TDHF, TDB3LYP, and TDCAM-B3LYP methodologies, are shown in Table 2. Analysis of the optical characteristics showed HOMO to LUMO transitions for all five molecules (except for Molecule 1, in B3LYP the observed transition was from HOMO-1 to LUMO, which is indicating donor to acceptor charge transfer during their excitations for each molecule natural transition orbitals, NTOs for Molecules 4 and 5 were analyzed and are provided in supporting information as Fig. S2. Qualitatively, it depicts the charge transfer characteristics in these molecules and especially in the case of Molecule 5, HOMO is completely localized in the donor side of the molecule). Overall, in all three methodologies, increasing trend in *λ*_max_ was observed. Only exceptions observed were between Molecules 3 and 4 in B3LYP methodology, where instead of an increasing *λ*_max_ value on moving from Molecule 3 to 4, a decreasing trend was observed. Except for Molecule 5, in HF method, all other case good absorption oscillator strengths were observed.

**Table 2:** Transition dipole moment (Δ*μ*_*ge*_) in Debye, excitation energies (Δ*E*_*ge*_) in eV, absorption maxima (*λ*_max_) in nm, oscillator strengths *f*_*ge*_ (unitless), coefficients of maximum contributing transitions *C*_*i*_ (unitless), and orbitals participating in the major transitions, for Molecules 1–5, computed using TDHF, TDB3LYP, and TDCAM-B3LYP methodologies

Molecule #	Methodologies	Δ*μ*_*ge*_	Δ*E*_*ge*_	*λ*_max_	Major transition	*C*_*i*_	*f*_*ge*_
1	HF	1.5	3.42	362.3	HOMO → LUMO	0.68	1.12
	B3LYP	9.6	3.08	402.5	HOMO-1 → LUMO	0.66	0.67
	CAM-B3LYP	10.4	3.20	387.3	HOMO → LUMO	0.70	0.92
2	HF	36.0	2.54	487.5	HOMO → LUMO	0.66	1.92
	B3LYP	17.4	2.24	551.8	HOMO → LUMO	0.73	1.40
	CAM-B3LYP	19.6	2.3	536.7	HOMO → LUMO	0.70	1.60
3	HF	9.6	2.07	597.4	HOMO → LUMO	0.65	1.56
	B3LYP	18.0	0.96	1280.0	HOMO → LUMO	0.76	1.58
	CAM-B3LYP	31.8	1.60	775.0	HOMO → LUMO	0.71	1.79
4	HF	74.9	1.70	727.5	HOMO → LUMO	0.69	0.28
	B3LYP	34.5	1.13	1094.3	HOMO → LUMO	0.84	1.47
	CAM-B3LYP	48.1	0.97	1278.0	HOMO → LUMO	0.75	1.28
5	HF	92.6	1.40	890.4	HOMO → LUMO	0.70	0.03
	B3LYP	42.7	0.73	1682.5	HOMO → LUMO	1.01	1.03
	CAM-B3LYP	62.8	0.53	2336.7	HOMO → LUMO	0.90	0.73

As expected for extended conjugated molecular systems, on moving from Molecule 1 to 5, Δ*E*_*ge*_ (optical gaps) values showed decreasing trend, thus resulting increasing trend in the *λ*_max_ values (as they are inversely proportional to each other). In other words, on moving from Molecule 1 to 5, absorptions were found to be red-shifted. It is to be noted here from Molecule 3 onwards (after the introduction of two benzene rings as the bridge) absorptions were observed in the IR regions of the electromagnetic spectrum (with CAM-B3LYP/6–31++G(d,p), Molecules 4 and 5 showed absorptions with *λ*_max_ = 1341.4 nm and *λ*_max_ = 2501.8 nm respectively and were red-shifted compared to their respective 6-31G(d,p) cases. Also, both showed intense HOMO to LUMO transitions) and showed absorption in the IR region (maybe helpful to solve transparency-tradeoff problem existing in NLO [79–83]). Comparing Δ*E*_*ge*_ values (optical gaps) with HLGs (electronic gaps), all the five molecules showed lower optical gaps and larger electronic gaps. Similar trends were also observed with HF and B3LYP methodologies. Like the observed trends of HLG, HF showed larger and B3LYP showed lower Δ*E*_*ge*_ values than CAM-B3LYP and thus as a result while HF showed lower values of *λ*_max_, at the same time B3LYP showed larger *λ*_max_ values.

### Polarizabilities and hyperpolarizabilities

From the optical absorption data discussed in the previous section, according to the two-state approximation (TSA) model (Eq. 5) [84, 85], with the sequential decreasing of the Δ*E*_*ge*_ values, exceptionally large values of hyperpolarizabilities can be expected on moving from Molecule 1 → 2 → 3 → 4 → 5. Note that Δ*E*_*ge*_ being a cubic term and placed in the denominator (Eq. 5), a slightest decrease in its value can result in exceptionally large hyperpolarizability.

5 $${\beta }^{TSA}=\frac{3}{5}\frac{{f}_{ge}{\Delta \mu }_{ge}}{{\Delta E}_{ge}^{3}}$$

where *f*_*ge*_ is the oscillator strength accompanying ground to first excited state transition, Δ*μ*_*ge*_ = *μ*_*e*_ − *μ*_*g*_ is the state dipole difference between the ground state and excited states (here in our cases, excited state transition dipole moments were considered instead of the actual state dipole moment of the excited state), and Δ*E*_*ge*_ is the excitation energy (being the cubic term and denominator, a slight change can impact significant changes in the *β* value).

With TSA model (fitting the CAM-B3LYP computed spectroscopic parameters from Table 2), computed hyperpolarizabilities (*β*) were found to be (in ×10^−30^ esu) 30.0, 264.7, 1424.2, 6929.0, and 31,629.0 (very large values of hyperpolarizabilities were observed), respectively for Molecules 1, 2, 3, 4, and 5. Interestingly, completely different behaviors were observed contrary to the expectation and are discussed in detail below (Table 3).

**Table 3:** Components of polarizabilities (*α*_*ii*_) and average polarizabilities (<*α*>) in ×10^−24^ esu, and total 1 st hyperpolarizabilities (*β*_0_) and major component (*β*_*xxx*_) in ×10^−30^ esu, of Molecules 1–5 from all three methodologies

Molecule #	Methodologies	*α*_*xx*_	*α*_*yy*_	*α*_*zz*_	<*α*>	*β*_*xxx*_	*β*_0_
1	HF	370.1	129.0	47.3	54.9	71.9	68.8
	B3LYP	382.0	138.3	47.6	56.6	28.9	26.6
	CAM-B3LYP	375.0	135.4	47.3	55.6	42.8	40.3
2	HF	894.3	178.6	65.5	132.5	508.9	500.8
	B3LYP	924.0	191.5	65.9	136.8	126.4	121.6
	CAM-B3LYP	930.4	187.2	65.5	137.9	259.2	252.8
3	HF	1237.8	222.3	98.5	183.4	2080.0	2070.1
	B3LYP	1875.7	244.5	87.1	278.0	320.9	314.3
	CAM-B3LYP	1953.6	239.4	86.2	289.5	1227.5	1216.8
4	HF	1025.5	255.4	145.2	152.0	1803.8	1797.9
	B3LYP	3347.4	273.8	135.2	496.0	380.2	373.4
	CAM-B3LYP	3887.5	265.8	137.7	576.1	3772.3	3760.4
5	HF	997.8	268.5	209.7	147.9	930.9	926.6
	B3LYP	5290.4	271.4	217.9	784.0	211.8	205.3
	CAM-B3LYP	6871.1	261.0	223.4	1018.3	2495.6	2486.0

Polarizabilities and hyperpolarizabilities were computed for Molecules 1–5 using all three methodologies (related tensorial components, from CAM-B3LYP, for all five molecules are provided in supporting information). Table 3 shows the major components of polarizabilities and hyperpolarizabilities, along with average polarizabilities (<*α*>) and total 1 st hyperpolarizabilities (*β*_0_). As expected on moving from Molecule 1 to 5, an increase in trend in average polarizabilities (<*α*>), in all three methodologies, was observed. While for Molecule 1, all three methodologies predicted nearly equal values of polarizabilities, for all other molecules while HF showed lower values, at the same time CAM-B3LYP showed larger values, and B3LYP showed intermediate values (closer to CAM-B3LYP values) of polarizabilities. Also, it is interesting to note that the largest value of polarizability in HF method was observed for Molecule 3 and thus Molecules 4 and 5 showed lower values of <*α*> than Molecule 3 in HF method. Analysis of the components of polarizabilities, from the three components (*α*_*xx*_, *α*_*yy*_, and *α*_*zz*_), *α*_*xx*_ was found to be showing much larger values than both *α*_*yy*_ and *α*_*zz*_ (between *α*_*yy*_ and *α*_*zz*_, *α*_*zz*_ showed much smaller values). But, it is to be noted here that with CAM-B3LYP/6–31++G(d,p), much lower values of polarizabilities were observed (Molecule 4: 224.9 × 10^−24^ esu and Molecule 5: 392.3 × 10^−24^ esu).

About the nonlinear optical property (the 1 st hyperpolarizability, *β*) contrary to the expectation (as expected according to the two-state model), rather a saturation limit for *β* was observed. While HF showed the saturation of *β* for Molecule 3, at the same time both B3LYP and CAM-B3LYP extended the saturation limit of *β* to the Molecule 4 (*n* = 3 or three benzene rings as bridges). Once the saturation limit for *β* was attained, then it started to decline, thus clearly indicating the unsuitability of two-state model in predicting the nonlinear optical behaviors of extremely conjugated systems beyond certain length. About the performances of methodologies, for all the molecules B3LYP predicted lowest values of *β* than the other two methodologies. At the same time up to Molecule 3, HF showed larger values of *β* than CAM-B3LYP, then a reverse trend was observed for Molecules 4 and 5. Thus, till Molecule 3, largest values of *β* were observed with HF and then with CAM-B3LYP. CAM-B3LYP computed *β* for Molecule 4 is the highest in the series. Molecule 1 showed near equal value of *β* for HF and CAM-B3LYP. For HF and B3LYP, the highest value of *β* observed were 2070.1 × 10^−30^ esu (Molecule 3) and 373.4 × 10^−30^ esu (Molecule 4) respectively. With CAM-B3LYP/6-31G(d,p), for Molecule 4, the highest value of *β* observed was 3760.4 × 10^−30^ esu and |*μβ*| (using Eq. 5) value around 180,875.2 × 10^−48^ esu (with CAM-B3LYP/6–31++G(d,p), the *μ*_*T*_ = 50.4 D, <*α*> = and *β*_0_ =). It is to be noted here that such large values are among the some of the largest values reported in the literature till date, for molecular chromophores [24–28, 31]. But, it is to be noted here that with CAM-B3LYP/6–31++G(d,p), enhanced values of hyperpolarizabilities were observed: Molecule 4: 5480.1 × 10^−30^ esu and Molecule 5: 4152.8 × 10^−30^ esu. Also, comparing both values, we can say that saturation of hyperpolarizability may be happening at Molecule 4, like discussed earlier.

### Possible pseudo-Jahn-Teller effect

Based on the continuous decrease in HLG and rigid structural topographies observed for the molecular systems considered here, Marder’s proposed “bond length alternation” [86, 87] and Dahne-Redaglia’s “cyanine limit” [88] cannot be applied to such zwitterions. Absence of these two effects (no bond length alternation was observed and HLG was found to be showing continually decreasing trend) was clearly observed for these molecules and hence cannot be used as an answer to the observed saturation limit for the molecular hyperpolarizabilities. But, moving from Molecule 1 (which is highly symmetric with C_2V_ molecular symmetry) to other molecules in the series, a systematic pattern of twisting, thus resulting in reduction of symmetry from C_2V_ to pure rotation C_2_ symmetry, was observed. Also, when orbitals (HOMO and LUMO) were analyzed (Fig. 2), one can say that depleted electron densities (localized rather than delocalized densities) in the central region was observed for Molecule 5. It is also to be noted that, instead of Molecule 5, saturation limits of hyperpolarizabilities were observed for Molecule 4 (HF showed even an early saturation at Molecule 3). As described by Tolbert and Zhao [89], a pseudo-Jahn-Teller effect (known otherwise as Peierls distortion [90]) can be expected at Molecule 5 and hence using that such a saturation limit can be explained. But, here the observed saturation conundrum can be an early effect of the Peierls distortion and thus for these Brooker’s type zwitterions, one can say that the saturation of molecular 1 st hyperpolarizability happens before the pseudo-Jahn-Teller effect.

**Fig. 3:**
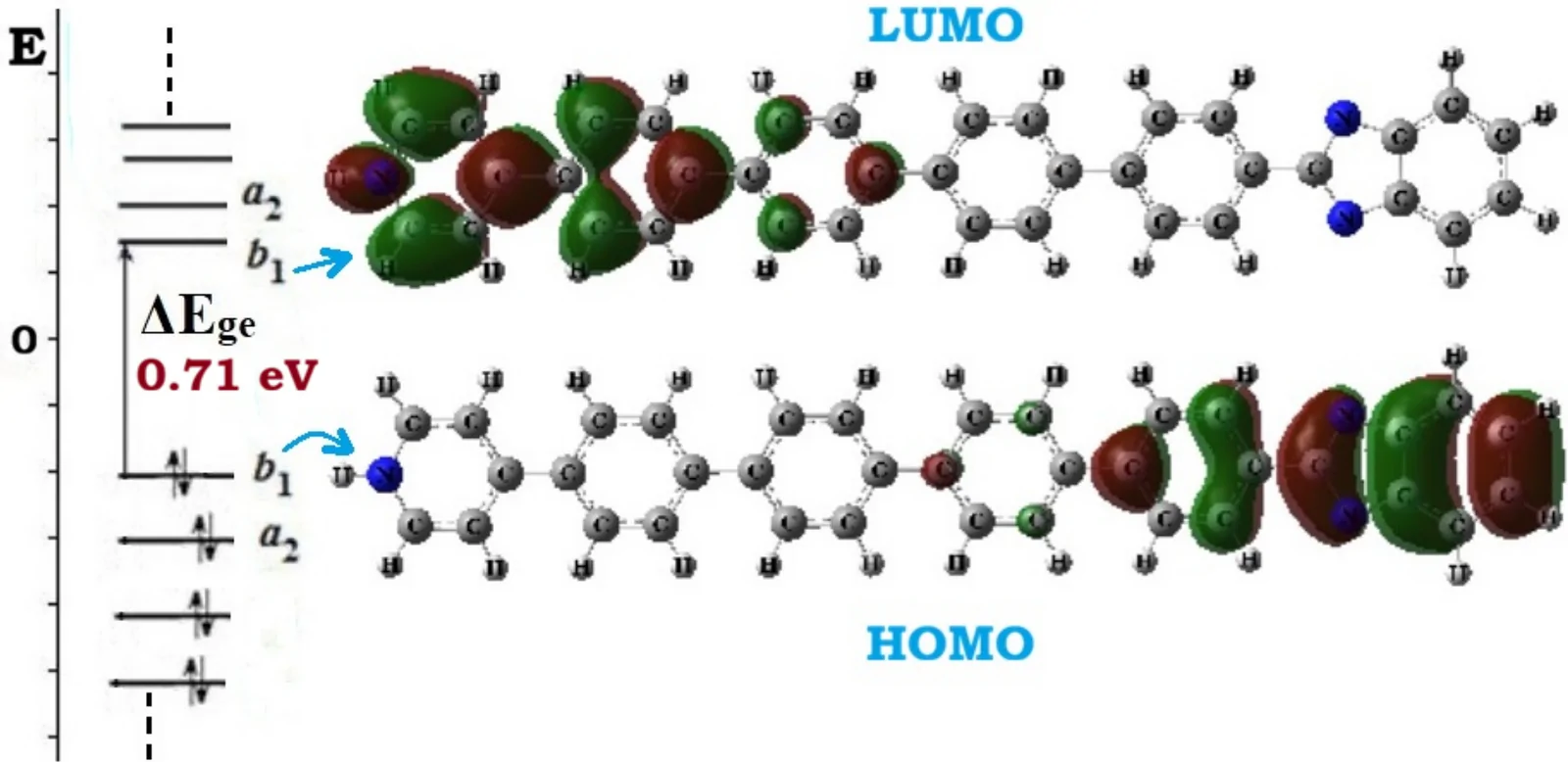
Representative diagram showing the vertical excitations and participating molecular orbitals of Molecule 5P (fully planar), computed using CAM-B3LYP/6-31G(d,p)

Hence, to check the possibility of pseudo-Jahn-Teller (PJT) effect, we selected Molecules 4 and 5 and investigated them with further computations. We optimized their fully planar conformations (now are Molecule 4P and Molecule 5P—optimized geometric conformations provided in supporting information—Fig. S3) with full C_2V_-point group symmetry considerations (using CAM-B3LYP/6-31G(d,p) method). As expected, Molecules 4P and 5P were found to be energetically less stable compared to their twisted counterparts, i.e., Molecules 4 and 5 respectively. While Molecule 4P showed three, at the same time Molecule 5P showed four imaginary frequencies (negative Eigen values in the Hessian; Molecule 4P: 69.6 *i* cm^−1^, 37.4 *i* cm^−1^, and 9.9 *i* cm^−1^, and Molecule 5P: 90.0 *i* cm^−1^, 68.2 *i* cm^−1^, 40.1 *i* cm^−1^, and 16.3 *i* cm^−1^). Both Molecules 4P and 5P were found to be in ^1^A_1_ states, with C_2V_-point group symmetry. Computed response properties for Molecule 4P were found to be *μ*_*T*_ = 45.7 Debye, <*α*> = 203.3 × 10^−24^ esu, and *β*_0_ = 3581.1 × 10^−30^ esu. Similarly, for Molecule 5P they were found to be *μ*_*T*_ = 60.5 Debye, <*α*> = 349.3 × 10^−24^ esu, and *β*_0_ = 4623.5 × 10^−30^ esu. Thus, showing the increasing trend for *β*_0_ with the planar conformations (no indication of saturation of hyperpolarizability), but for twisted conformation the saturation was observed for Molecule 4. Now to qualitatively analyze the possible pseudo-Jahn-Teller effect, excitations and orbital computations were carried out. Excitations of Molecule 5P and its corresponding participating orbitals were shown in Fig. 3 (for Molecule 4, orbitals are provided in supporting information—Fig. S4). From the excitation analysis, it was observed that Molecule 4P, when excited to first excited state via HOMO to LUMO transition, excitation energy (Δ*E*_*ge*_) was found to be 1.13 eV (*λ*_max_ = 1100.5 nm) with *f*_*ge*_ = 1.6461. Similarly, Molecule 5P, when excited to first excited state via HOMO to LUMO transition, excitation energy (Δ*E*_*ge*_) was found to be 0.71 eV (*λ*_max_ = 1744.1 nm) with *f*_*ge*_ = 1.2373.

At first when the respective imaginary frequencies of Molecules 4P and 5P were analyzed, it was observed that all those frequencies were related to inter-ring twistings in respective cases. This shows that, in both the molecules, with possible symmetry breaking on moving from C_2V_ molecular symmetry to a pure rotational C_2_ symmetry, both molecules will be able to produce their respective minimum energy conformations (the twisted conformations). In fact, as expected, it was also observed that Molecules 4 and 5 were found to be true minima compared to Molecules 4P and 5P respectively. Now analysis of the molecular orbitals of Molecule 4P, one can clearly see delocalized HOMO and LUMO (supporting information), and it was definitely expected owing to its planar conformation. However, a similar situation was not observed for Molecule 5P and in spite of its planar conformation (which can facilitate the delocalization), still localized HOMO and LUMO were observed (Fig. 3). Now considering the excitation and related MO diagrams of Molecule 5P (Fig. 3), the first excited state was found to be from HOMO to LUMO (b_1_ → b_1_ symmetry allowed transition). It is also to be noted that this excited state is of A_1_ symmetry (so also its ground state, which is also in A_1_ state). Due to such favorable symmetric conditions, in case of Molecule 5P, one can expect the possibility of vibronic mixing of its ground state and excited state, through its b_1_ distortion modes (which is of rotational in nature in our case) [91]. Now, comparing the HOMO and LUMO of Molecule 5P (Fig. 3), with their respective reduced symmetry counterparts, Molecule 5 (Fig. 2), one can clearly see the mixing of the orbitals in the high symmetry state to generate the orbitals of low symmetry state (Fig. S5—supporting information). Thus, with this analysis, we can say that only in case of Molecule 5P, both HOMO and LUMO can be regarded as responsible for pseudo-Jahn-Teller effect, leading to the reduced symmetry twisted conformation.

## Conclusion

Current work reports systematic studies focusing on structure and nonlinear optical properties of a series of Brooker’s types of zwitterionic molecules with varied number of aromatic benzene rings as the bridges. As reported, without any bridge (Molecule 1), dominant forces were hydrogen bonding interactions, thus imposing molecular planarity. Although hydrogen bonding interactions were present in all other molecules considered here, as soon as a benzene ring was introduced as the bridge, immediately the competition between destabilizing repulsive steric forces (which demands twisted conformation) and stabilizing resonance forces (which demands planar conformation) can be expected to have started, and steric forces might be dominating over resonance forces. Conformational switching from planar to twisted conformation started from Molecule 2, but a major switchover was observed in Molecule 3 and in latter molecules (from the molecular structures, one can see that the number of steric repulsions increases with each successive molecule). Now about the properties, while HLG showed decreasing trends, at the same time *λ*_max_ showed increasing trend (red shifting). Also, both the dipole moments and the polarizabilities were found to be showing linearly increasing trends. Interestingly, hyperpolarizabilities showed clearly a nonlinear trend, with the saturation point at Molecule 4. Nevertheless, while pseudo-Jahn-Teller distortion was observed for Molecule 5, at the same time, Molecule 4 showed the *β* to be 3760.4 × 10^−30^ esu, indicating an optimal twisted conformation for saturation of hyperpolarizability, before Peierls distortion. It is to be noted here that such a large value is one among the few largest values of *β* reported in literature. Also, with the introduction of more than one benzene rings as the bridges, such types of zwitterions showed absorption in the IR region, thus may be helpful in providing solutions to the transparency-tradeoff problem of nonlinear optics. Also, they can be used as NIR dyes for other fundamental applications. In conclusion, with the observed structure-property correlations for these extended conjugated zwitterions, not only can they be considered useful in the field of nonlinear optics but also could possibly find applications in other areas of functional material science.

## Supplementary Information

Below is the link to the electronic supplementary material.

ESM 1 DOCX (1.93 MB)

## Data Availability

No datasets were generated or analysed during the current study.
