# Supplementary material for: Competition among steric vs resonance forces and conformational switchover: possible pseudo-Jahn-Teller effects and saturation of hyperpolarizabilities in Brooker’s zwitterions
Source: J Mol Model. 2026 Nov 3;32(11):455. doi: 10.1007/s00894-026-07040-8 (PMC13731493; doi:10.1007/s00894-026-07040-8)
Supplement: Supplementary file 1 [file 894_2026_7040_MOESM1_ESM.docx]

**Competition Among Steric vs Resonance Forces and Conformational Switchover: Possible Pseudo Jahn-Teller Effects and Saturation of Hyperpolarizabilities in Brooker’s Zwitterions**

Divya Pant, Bharati Bamana, Sanyasi Sitha*

Department of Chemical Sciences, University of Johannesburg, PO Box 524, Auckland Park, Johannesburg, South Africa 2006.

**Figure S1:** Molecular electrostatic potential (MEP or ESP) maps of Molecules 1-5, computed using CAM-B3LYP/6-31G(d,p) optimized geometries and in the same methodology.

**Figure S2:** HOMOs and LUMOs (all are natural transition orbitals) of Molecule 4 and 5 (both twisted conformations), computed using CAM-B3LYP/6-31G(d,p) method. (Iso values: 0.02)

**Figure S3:** Fully planar minimum energy conformations of Molecules 4P and 5P, computed using CAM-B3LYP/6-31G(d,p).

**Figure S4:** HOMO and LUMO of Molecule 4P (planar), computed using CAM-B3LYP/6-31G(d,p) method. (Iso values: 0.02)

**Figure S5:** HOMO and LUMO of Molecule 5P (planar – left side), mixing to produce HOMO and LUMO of Molecule 5 (twisted – right side), computed using CAM-B3LYP/6-31G(d,p) method. (Iso values: 0.02)

Components of dipole moments (in Debye) of Molecules 1 - 5

| Molecule No. | μx | μy | μz |
| --- | --- | --- | --- |
| 1 | -10.30 | 0 | 0 |
| 2 | 19.62 | 0 | 0 |
| 3 | -31.77 | 0 | 0 |
| 4 | 48.08 | 0 | 0 |
| 5 | -62.84 | 0 | 0 |

Three major components (in au) polarizabilities values of Molecules 1 - 5

| Molecule No. | αxx | αyy | αzz |
| --- | --- | --- | --- |
| 1 | 375.013 | 135.353 | 47.289 |
| 2 | 930.429 | 187.176 | 65.527 |
| 3 | 1953.657 | 239.398 | 86.235 |
| 4 | 3887.463 | 265.809 | 137.665 |
| 5 | 6871.146 | 260.994 | 223.037 |

Nine components of hyperpolarizabilities (in au) of Molecules 1 - 5

| Molecule No. | 1 | 2 | 3 | ~~4~~ | 5 |
| --- | --- | --- | --- | --- | --- |
| βxxx | -4955.929 | 30003.14 | -142077.70 | 436647.85 | -288861.24 |
| βxxy | 0.00009138 | -0.003574 | -0.0043745 | -0.036559 | -0.0010484 |
| βxyy | 296.511 | -755.908 | 1242.663 | -1145.617 | 808.88761 |
| βyyy | 0.00009548 | 0.0007227 | -0.0007193 | 0.0003819 | 0.0000101 |
| βxxz | 0.0094893 | 0.0000419 | 0.078674 | -0.091039 | 0.0027405 |
| βyyz | 0.00007129 | 0.0000429 | -0.000698 | 0.0004350 | -0.000014 |
| βxzz | -5.82771 | 13.45175 | -5.24670 | -240.7178 | 296.67470 |
| βyzz | 0.00001512 | -0.0000210 | 0.000691 | 0.0004433 | 0.0000150 |
| βzzz | -0.00050899 | 0.00007589 | -0.000091 | 0.0007040 | -0.0000294 |

**First six harmonic frequencies of Molecules 1-5, from CAM-B3LYP/6-31G(d,p) computations.**

**Molecule 1:**

```
                     1                      2                      3
                     A                      A                      A
 Frequencies --    61.7344                87.8686               109.0776
 Red. masses --     5.7627                 3.8823                 4.9196
 Frc consts  --     0.0129                 0.0177                 0.0345
 IR Inten    --     7.5375                 0.0000                 4.3824
 Raman Activ --     0.0005                 0.0002                 0.0077
 Depolar (P) --     0.7500                 0.7500                 0.7500
 Depolar (U) --     0.8571                 0.8571                 0.8571

                     4                      5                      6
                     A                      A                      A
 Frequencies --   174.4419               280.9915               301.2226
 Red. masses --     4.8348                 5.5914                 6.7851
 Frc consts  --     0.0867                 0.2601                 0.3627
 IR Inten    --     0.0191                 0.0000                 8.1500
 Raman Activ --     3.2404                 0.8862                44.3905
 Depolar (P) --     0.7500                 0.7500                 0.3079
 Depolar (U) --     0.8571                 0.8571                 0.4708
```

**Molecule 2:**

```
                     1                      2                      3
                     A                      A                      A
 Frequencies --    21.3539                28.1134                66.9910
 Red. masses --     3.5663                 6.0791                 5.6852
 Frc consts  --     0.0010                 0.0028                 0.0150
 IR Inten    --     0.0000                 2.6140                 2.1291
 Raman Activ --     0.0004                 0.0420                 0.1799
 Depolar (P) --     0.7471                 0.7500                 0.7500
 Depolar (U) --     0.8552                 0.8571                 0.8571

                     4                      5                      6
                     A                      A                      A
 Frequencies --    81.9274                83.9245               144.4470
 Red. masses --     6.0165                 3.6974                 4.6734
 Frc consts  --     0.0238                 0.0153                 0.0575
 IR Inten    --     0.0577                 0.0000                 3.0187
 Raman Activ --     4.0226                 0.0049                 0.0041
 Depolar (P) --     0.7500                 0.7499                 0.7500
 Depolar (U) --     0.8571                 0.8571                 0.8571
```

**Molecule 3:**

```
                     1                      2                      3

                     A                      A                      A
 Frequencies --    16.3188                29.0309                38.5109
 Red. masses --     6.3709                 3.8356                 6.0454
 Frc consts  --     0.0010                 0.0019                 0.0053
 IR Inten    --     1.1998                 0.0002                 0.7346
 Raman Activ --     0.3934                 1.8593                 2.1713
 Depolar (P) --     0.7500                 0.4214                 0.7500
 Depolar (U) --     0.8571                 0.5929                 0.8571

                     4                      5                      6
                     A                      A                      A
 Frequencies --    47.8672                51.7350                87.4527
 Red. masses --     3.4599                 6.2626                 3.6470
 Frc consts  --     0.0047                 0.0099                 0.0164
 IR Inten    --    12.0564                 0.2895                 8.4301
 Raman Activ --    24.5001                 3.9241                23.5561
 Depolar (P) --     0.2792                 0.7500                 0.2824
 Depolar (U) --     0.4365                 0.8571                 0.4404
```

**Molecule 4**

```
                 1                      2                      3
                       A                      A                      A
 Frequencies -- 11.2896                21.5153             23.1248
 Red. masses --  6.5886                 6.4135              3.9240
 Frc consts  --  0.0005                 0.0017              0.0012
 IR Inten    --  0.6797                 0.7623              7.2278
 Raman Activ --  1.8594                 6.1373            204.9235
 Depolar (P) --  0.7500                 0.7500              0.3431
 Depolar (U) --  0.8571                 0.8571              0.5109

                 4                      5                      6
                 A                      A                      A
 Frequencies -- 42.1960               49.8331              52.3959
 Red. masses --  6.4285                3.7464               6.2996
 Frc consts  --  0.0067                0.0055               0.0102
 IR Inten    --  0.0987                2.2730               1.0960
 Raman Activ --  4.7437               11.9402               3.5058
 Depolar (P) --  0.7500                0.3981               0.7500
 Depolar (U) --  0.8571                0.5695               0.8571
```

**Molecule 5**

```
                     1                      2                      3
                     A                      A                      A
 Frequencies --     8.5883                12.7698                22.0036
 Red. masses --     6.7617                 6.6812                 4.0102
 Frc consts  --     0.0003                 0.0006                 0.0011
 IR Inten    --     0.4158                 0.6856                 1.3393
 Raman Activ --     3.5584                 9.0905                90.3870
 Depolar (P) --     0.7500                 0.7500                 0.4171
 Depolar (U) --     0.8571                 0.8571                 0.5886

                     4                      5                      6
                     A                      A                      A
 Frequencies --    31.7696                35.7016                45.4880
 Red. masses --     6.4845                 6.5614                 3.8526
 Frc consts  --     0.0039                 0.0049                 0.0047
 IR Inten    --     0.6885                 0.2192                 3.8904
 Raman Activ --    26.9324                13.2602                10.1201
 Depolar (P) --     0.7500                 0.7500                 0.7452
 Depolar (U) --     0.8571                 0.8571                 0.8540
```

**CAM-B3LYP/6-31G(d,p) optimized geometries in cartesian coordinates, for Molecules 1-5. Please refer to the main text of the article for molecule numbering and visuals of their minimum energy conformations.**

**Molcule-1**

X	Y	Z

C	-0.00099500	1.21582500	2.34311200

C	0.00099500	-1.21582500	2.34311200

C	-0.00092400	1.18778000	3.70250800

H	-0.00208800	2.15356600	1.80222900

C	0.00092400	-1.18778000	3.70250800

H	0.00208800	-2.15356600	1.80222900

H	-0.00127800	2.08049600	4.31546400

H	0.00127800	-2.08049600	4.31546400

H	0.00000000	0.00000000	5.38113900

C	0.00000000	0.00000000	1.60256600

N	0.00000000	0.00000000	4.37161600

C	0.00000000	0.00000000	0.18817000

C	0.00016500	-0.71951900	-1.79298600

C	-0.00016500	0.71951900	-1.79298600

C	0.00036800	-1.43755500	-3.01514800

C	-0.00036800	1.43755500	-3.01514800

C	0.00026500	-0.71534700	-4.18009100

H	0.00068000	-2.52271200	-3.01465300

C	-0.00026500	0.71534700	-4.18009100

H	-0.00068000	2.52271200	-3.01465300

H	0.00036100	-1.23351200	-5.13437100

H	-0.00036100	1.23351200	-5.13437100

N	0.00000000	1.15954700	-0.52521100

N	0.00000000	-1.15954700	-0.52521100

**Molecule 2**

X	Y	Z

C	-2.39477221	-0.57489816	0.18685729

C	-0.99837401	-0.57478953	-0.18777449

C	-0.32366509	0.61570295	-0.36890380

C	-1.02542930	1.81813637	-0.18061348

C	-2.36788759	1.81803095	0.17966821

C	-3.06950442	0.61551137	0.36802283

C	-1.69670232	-2.78004288	-0.00054343

H	0.73755299	0.63231849	-0.65376580

H	-0.49819721	2.77341939	-0.32213354

H	-2.89527454	2.77319895	0.32131164

H	-4.13068731	0.63213084	0.65292912

C	-1.69648233	-4.32004285	-0.00072087

C	-0.48810491	-5.01739165	-0.00102125

C	-2.90428746	-5.01772104	-0.00047010

C	-0.48762799	-6.41210344	-0.00175270

H	0.46393429	-4.46706249	-0.00199614

C	-2.90398345	-6.41285892	-0.00020578

H	-3.85676909	-4.46825985	-0.00005344

C	-1.69593514	-7.11010759	-0.00098513

H	0.46478804	-6.96183017	-0.00262351

H	-3.85643847	-6.96268006	0.00040734

C	-2.86188464	-9.34814588	-0.31432813

C	-0.52776966	-9.34709522	0.31004729

C	-2.86150650 -10.74285788	-0.31450523

H	-3.78218858	-8.79836126	-0.55931653

C	-0.52696664 -10.74223250	0.30895279

H	0.39200666	-8.79709009	0.55628888

H	-3.78133670 -11.29312883	-0.56029130

H	0.39364503 -11.29150911	0.55439846

H	-1.69353413 -12.43017120	-0.00335259

N	-2.80711645	-1.96864925	0.29737985

N	-0.58588257	-1.96831646	-0.29845238

N	-1.69359887 -11.44017123	-0.00311736

C	-1.69505844	-8.65010717	-0.00171014

**Molecule 3**

X	Y	Z

C	-2.36421695	-0.57491754	0.27685244

C	-1.02881210	-0.57477027	-0.27719700

C	-0.38362659	0.61574088	-0.54502375

C	-1.05477410	1.81815488	-0.26657451

C	-2.33857924	1.81801231	0.26619328

C	-3.00949613	0.61547332	0.54470264

C	-1.69658395	-2.78004294	-0.00022512

H	0.63121785	0.63238579	-0.96623623

H	-0.55061064	2.77345249	-0.47584214

H	-2.84294053	2.77316572	0.47559102

H	-4.02430094	0.63206343	0.96595422

C	-1.69634033	-4.32004291	-0.00041978

C	-0.49836008	-5.01735484	-0.15875743

C	-2.89371556	-5.01775795	0.15780293

C	-0.49793880	-6.41206662	-0.15953589

H	0.44535620	-4.46699664	-0.28424530

C	-2.89333545	-6.41289582	0.15803430

H	-3.83797828	-4.46832582	0.28278806

C	-1.69574413	-7.11010763	-0.00073516

H	0.44619950	-6.96176429	-0.28496270

H	-3.83751132	-6.96274601	0.28321789

C	-2.89258965	-9.34818482	-0.15887675

C	-0.49686229	-9.34705632	0.15485501

C	-2.89219380 -10.74289681	-0.15909271

H	-3.83704777	-8.79843088	-0.28139719

C	-0.49616517 -10.74219358	0.15367397

H	0.44720185	-8.79702051	0.27867972

C	-1.69356981 -11.44017122	-0.00312644

H	-3.83625177 -11.29319843	-0.28245876

H	0.44865782 -11.29143950	0.27660750

N	-2.75851975	-1.96868004	0.44036544

N	-0.63429549	-1.96828578	-0.44086509

C	-1.69492106	-8.65010719	-0.00155858

C	-0.57673627 -13.67772921	0.45780151

C	-2.80980842 -13.67764019	-0.46487247

C	-0.57617036 -15.07244095	0.45704877

H	0.30350302 -13.12756481	0.82076118

C	-2.80978282 -15.07277813	-0.46477114

H	-3.69012161 -13.12801410	-0.82831281

H	0.30425569 -15.62233250	0.82004431

H	-3.69026810 -15.62243437	-0.82822389

H	-1.69258175 -16.76023564	-0.00444486

N	-1.69317113 -15.77023590	-0.00404563

C	-1.69346909 -12.98017117	-0.00349235

**Molecule 4**

X	Y	Z

C	-8.14161600	0.70256300	-0.08195296

C	-8.14161600	-0.70256000	0.08197604

C	-9.35017900	-1.40690700	0.16416104

C	-10.52695800	-0.70016300	0.08170404

C	-10.52695800	0.70016700	-0.08167696

C	-9.35017900	1.40691000	-0.16413496

C	-6.17419400	-0.00000300	-0.00002196

H	-9.35031800	-2.47704500	0.28908204

H	-11.46943200	-1.22040800	0.14241804

H	-11.46943200	1.22041300	-0.14238696

H	-9.35031800	2.47704900	-0.28905396

C	-4.69975300	-0.00000200	-0.00001896

C	-3.98328800	-1.18851000	0.13958804

C	-3.98328800	1.18850600	-0.13962196

C	-2.60358500	-1.18777200	0.13527104

H	-4.53437000	-2.10205700	0.25699304

C	-2.60358600	1.18776900	-0.13529996

H	-4.53437000	2.10205200	-0.25703096

C	-1.88466000	-0.00000200	-0.00001296

H	-2.07893000	-2.11875900	0.26938704

H	-2.07893000	2.11875600	-0.26941496

C	0.32330800	1.01559400	0.62602104

C	0.32331000	-1.01559700	-0.62603896

C	1.70281900	1.01571300	0.62772004

H	-0.20280300	1.80467500	1.13021304

C	1.70282100	-1.01571700	-0.62773296

H	-0.20279900	-1.80467900	-1.13023096

C	2.42034600	-0.00000100	-0.00000496

H	2.22298900	1.82654300	1.10805304

H	2.22299300	-1.82654700	-1.10806296

N	-6.85556500	1.13260200	-0.13214396

N	-6.85556500	-1.13259900	0.13217004

C	-0.40408000	-0.00000100	-0.00001096

C	4.62173100	-0.38267800	-1.13513696

C	4.62172600	0.38267600	1.13513504

C	6.00249100	-0.39096300	-1.14131696

H	4.09381000	-0.66277700	-2.02681796

C	6.00248600	0.39096400	1.14132104

H	4.09380100	0.66277600	2.02681404

C	6.67806300	0.00000100	0.00000304

H	6.54060000	-0.69907400	-2.02054596

H	6.54059100	0.69907500	2.02055204

C	3.90116400	-0.00000200	-0.00000196

C	8.79548000	-0.87874000	0.76478204

C	8.79548200	0.87874300	-0.76476796

C	10.16758800	-0.89961700	0.79198704

H	8.18895100	-1.55392700	1.33455004

C	10.16759000	0.89962100	-0.79196796

H	8.18895500	1.55392800	-1.33453896

H	10.67283400	-1.61275400	1.41364804

H	10.67283800	1.61275700	-1.41362796

H	11.85540100	0.00000200	0.00001196

C	8.13458800	0.00000200	0.00000704

N	10.86540100	0.00000200	0.00001104

**Molecule 5**

X	Y	Z

C	10.30281600	-0.71301100	-0.06848304

C	10.30281600	0.71301200	0.06847096

C	11.51665800	1.42005200	0.13712796

C	12.69861400	0.70464300	0.06822696

C	12.69861400	-0.70464100	-0.06824704

C	11.51665800	-1.42005100	-0.13714404

C	8.32368900	0.00000000	0.00000196

H	11.51598900	2.50183300	0.24311696

H	13.65003700	1.22891900	0.11988596

H	13.65003700	-1.22891700	-0.11991104

H	11.51599000	-2.50183100	-0.24313404

C	6.85971900	0.00000000	0.00000496

C	6.14053200	1.20012900	0.09649696

C	6.14053200	-1.20013000	-0.09648604

C	4.75585200	1.19914300	0.09211196

H	6.70590800	2.12192600	0.18244596

C	4.75585200	-1.19914300	-0.09209804

H	6.70590800	-2.12192700	-0.18243604

C	4.03266900	0.00000000	0.00000796

H	4.22328200	2.14118800	0.19819296

H	4.22328200	-2.14118800	-0.19817804

C	1.82822000	-1.03453400	0.60563396

C	1.82822100	1.03453400	-0.60561904

C	0.44260300	-1.03567500	0.60635796

H	2.36259000	-1.84124800	1.09679796

C	0.44260400	1.03567500	-0.60634404

H	2.36259100	1.84124800	-1.09678304

C	-0.27936000	0.00000000	0.00000596

H	-0.08631600	-1.86542300	1.06734796

H	-0.08631500	1.86542300	-1.06733504

N	9.01551100	-1.15366000	-0.11042704

N	9.01551100	1.15366100	0.11041496

C	2.55865000	0.00000000	0.00000796

C	-2.48169900	0.48291500	-1.09853504

C	-2.48170100	-0.48291700	1.09854396

C	-3.86702100	0.48275100	-1.10069204

H	-1.94766900	0.84873900	-1.96925204

C	-3.86702300	-0.48275200	1.10069796

H	-1.94767200	-0.84874100	1.96926096

C	-4.58625900	-0.00000100	0.00000196

H	-4.39380200	0.88627300	-1.96083404

H	-4.39380500	-0.88627400	1.96083896

C	-1.75497700	-0.00000100	0.00000496

C	-6.78392700	0.25485500	1.17612196

C	-6.78392400	-0.25485700	-1.17612204

C	-8.16973800	0.25160100	1.18709196

H	-6.24982700	0.46959400	2.09495696

C	-8.16973500	-0.25160200	-1.18709604

H	-6.24982200	-0.46959600	-2.09495604

C	-8.85105200	0.00000000	-0.00000304

H	-8.71019500	0.42398500	2.11278796

H	-8.71019000	-0.42398500	-2.11279304

C	-6.06047900	-0.00000100	0.00000096

C	-10.96728600	-1.05610000	-0.51887304

C	-10.96728700	1.05610200	0.51886296

C	-12.34593200	-1.07264600	-0.54079704

H	-10.35456000	-1.86973700	-0.88452004

C	-12.34593300	1.07264900	0.54078396

H	-10.35456100	1.86973800	0.88451096

H	-12.85428900	-1.92998100	-0.96357204

H	-12.85429000	1.92998400	0.96355696

H	-14.04152300	0.00000200	-0.00000896

C	-10.29705900	0.00000100	-0.00000404

N	-13.05152300	0.00000200	-0.00000804

**Molecule 4P**

C	0.00000000	0.71958400	-8.23005900

C	0.00000000	-0.71958400	-8.23005900

C	0.00000000	-1.43175200	-9.44883600

C	0.00000000	-0.71202400 -10.61868500

C	0.00000000	0.71202400 -10.61868500

C	0.00000000	1.43175200	-9.44883600

C	0.00000000	0.00000000	-6.25089800

H	0.00000000	-2.51731500	-9.44787800

H	0.00000000	-1.23306400 -11.57194800

H	0.00000000	1.23306400 -11.57194800

H	0.00000000	2.51731500	-9.44787800

C	0.00000000	0.00000000	-4.81282500

C	0.00000000	-1.20700100	-4.08627500

C	0.00000000	1.20700100	-4.08627500

C	0.00000000	-1.20295400	-2.71320700

H	0.00000000	-2.13245700	-4.64982900

C	0.00000000	1.20295400	-2.71320700

H	0.00000000	2.13245700	-4.64982900

C	0.00000000	0.00000000	-1.96875400

H	0.00000000	-2.15917700	-2.20380800

H	0.00000000	2.15917700	-2.20380800

C	0.00000000	0.00000000	-0.51632000

C	0.00000000	1.19560700	0.24063700

C	0.00000000	-1.19560700	0.24063700

C	0.00000000	1.19549300	1.61288800

H	0.00000000	2.15195600	-0.26486100

C	0.00000000	-1.19549300	1.61288800

H	0.00000000	-2.15195600	-0.26486100

C	0.00000000	0.00000000	2.36542700

H	0.00000000	2.15653100	2.11251700

H	0.00000000	-2.15653100	2.11251700

C	0.00000000	0.00000000	3.81207900

C	0.00000000	-1.19954200	4.56876300

C	0.00000000	1.19954200	4.56876300

C	0.00000000	-1.20076300	5.93741800

H	0.00000000	-2.15519000	4.06212100

C	0.00000000	1.20076300	5.93741800

H	0.00000000	2.15519000	4.06212100

C	0.00000000	0.00000000	6.69159000

H	0.00000000	-2.16213200	6.43678100

H	0.00000000	2.16213200	6.43678100

C	0.00000000	0.00000000	8.12007500

C	0.00000000	1.20312400	8.89203300

C	0.00000000	-1.20312400	8.89203300

C	0.00000000	1.18060800	10.25020100

H	0.00000000	2.17038900	8.41125700

C	0.00000000	-1.18060800	10.25020100

H	0.00000000	-2.17038900	8.41125700

H	0.00000000	2.08022400	10.85202000

H	0.00000000	-2.08022400	10.85202000

H	0.00000000	0.00000000	11.93635300

N	0.00000000	1.15940900	-6.95695100

N	0.00000000	-1.15940900	-6.95695100

N	0.00000000	0.00000000	10.92677800

**Molecule 5P**

C	0.00000000	0.72006300 -10.40815000

C	0.00000000	-0.72006300 -10.40815000

C	0.00000000	-1.43123000 -11.62554000

C	0.00000000	-0.71142900 -12.79718000

C	0.00000000	0.71142900 -12.79718000

C	0.00000000	1.43123000 -11.62554000

C	0.00000000	0.00000000	-8.42855000

H	0.00000000	-2.51686800 -11.62486600

H	0.00000000	-1.23332800 -13.75008100

H	0.00000000	1.23332800 -13.75008100

H	0.00000000	2.51686800 -11.62486600

C	0.00000000	0.00000000	-6.98200200

C	0.00000000	-1.20348500	-6.25774500

C	0.00000000	1.20348500	-6.25774500

C	0.00000000	-1.19930900	-4.88036700

H	0.00000000	-2.13006900	-6.81975500

C	0.00000000	1.19930900	-4.88036700

H	0.00000000	2.13006900	-6.81975500

C	0.00000000	0.00000000	-4.14053700

H	0.00000000	-2.15563200	-4.37074600

H	0.00000000	2.15563200	-4.37074600

C	0.00000000	0.00000000	-2.67262700

C	0.00000000	1.19075700	-1.92151400

C	0.00000000	-1.19075700	-1.92151400

C	0.00000000	1.19067300	-0.54308900

H	0.00000000	2.14791100	-2.42630100

C	0.00000000	-1.19067300	-0.54308900

H	0.00000000	-2.14791100	-2.42630100

C	0.00000000	0.00000000	0.20248200

H	0.00000000	2.15161700	-0.04283500

H	0.00000000	-2.15161700	-0.04283500

C	0.00000000	0.00000000	1.67030400

C	0.00000000	-1.19197600	2.41870800

C	0.00000000	1.19197600	2.41870800

C	0.00000000	-1.19257900	3.79617200

H	0.00000000	-2.14900300	1.91354300

C	0.00000000	1.19257900	3.79617200

H	0.00000000	2.14900300	1.91354300

C	0.00000000	0.00000000	4.54215400

H	0.00000000	-2.15366200	4.29552000

H	0.00000000	2.15366200	4.29552000

C	0.00000000	0.00000000	6.00347000

C	0.00000000	1.19610900	6.75378200

C	0.00000000	-1.19610900	6.75378200

C	0.00000000	1.19844600	8.12672900

H	0.00000000	2.15220300	6.24764700

C	0.00000000	-1.19844600	8.12672900

H	0.00000000	-2.15220300	6.24764700

C	0.00000000	0.00000000	8.87602400

H	0.00000000	2.16013900	8.62519800

H	0.00000000	-2.16013900	8.62519800

C	0.00000000	0.00000000	10.31438900

C	0.00000000	-1.20178500	11.08174100

C	0.00000000	1.20178500	11.08174100

C	0.00000000	-1.18110100	12.44166300

H	0.00000000	-2.16882600	10.60022700

C	0.00000000	1.18110100	12.44166300

H	0.00000000	2.16882600	10.60022700

H	0.00000000	-2.08041500	13.04373600

H	0.00000000	2.08041500	13.04373600

H	0.00000000	0.00000000	14.12627600

N	0.00000000	0.00000000	13.11664400

N	0.00000000	1.15862500	-9.13275200

N	0.00000000	-1.15862500	-9.13275200
